# Evaluation of the Xpert Carba-R assay for quantifying carbapenemase-producing bacterial load in stool samples

**DOI:** 10.1371/journal.pone.0309089

**Published:** 2024-08-28

**Authors:** Jie Yin Chua, Ze Qin Lim, Song Qi Dennis Loy, Vanessa Koh, Natascha May Thevasagayam, Xiaowei Huan, Kyaw Zaw Linn, Kalisvar Marimuthu, Oon Tek Ng

**Affiliations:** 1 National Centre for Infectious Diseases, Singapore, Singapore; 2 Tan Tock Seng Hospital, Singapore, Singapore; 3 Yong Loo Lin School of Medicine, National University of Singapore, Singapore, Singapore; 4 Lee Kong Chian School of Medicine, Nanyang Technological University, Singapore, Singapore; AIIMS: All India Institute of Medical Sciences, INDIA

## Abstract

**Background:**

The spread of Carbapenemase-producing Organisms (CPO) remains a major threat globally. Within clinical settings, the existing method of determining gene load involves traditional culture to determine bacterial load and polymerase-chain-reaction-based Xpert Carba-R Assay to determine carbapenemase gene type. However, there is a need for a fast and accurate method of quantifying CPO colonisation to study the risk of persistent CPO carriage.

**Objective:**

This study evaluated the accuracy of Xpert Carba-R Ct value in estimating carbapenamase producing bacterial loads in stool samples.

**Methods:**

Stool samples were obtained from an ongoing study investigating the household transmission of CPO in Singapore. Stool samples lacking carbapenemase producing organisms were spiked with organism carrying a single carbapenemase gene (*bla*_KPC_, *bla*_NDM_, *bla*_VIM_, *bla*_OXA-48(-like)_ or *bla*_IMP-1_) and serially diluted before being subjected to Xpert Carba-R assay and traditional culture. Standard curves with regression lines showing correlation between C_t_ values and plate counts were generated. The standard curves were validated with stool samples collected from patients.

**Results:**

The limit of detection of *bla*_NDM_, *bla*_KPC_, and *bla*_OXA-48_ was approximately 10^3^ cfu/mL, while that of *bla*_IMP-1_ and *bla*_VIM_ was approximately 10^4^ cfu/mL. Validation of the *bla*_NDM_ and *bla*_OXA-48_ curves revealed average delta values of 0.56 log(cfu/mL) (95% CI 0.24–0.88) and 0.80 log(cfu/mL) (95% CI 0.53–1.07), respectively.

**Conclusions:**

Our validation data for stool positive for *bla*_NDM_ and *bla*_OXA-48-type_ suggests that bacterial loads can be estimated within a reasonable range of error.

## Introduction

Conventional culture-based methods of detecting Carbepenemase-producing Organisms (CPO) are often time consuming, with variations in sensitivity and specificity affected by media composition [1, 2]. The development of the on-demand PCR-based Xpert Carba-R Assay has greatly reduced turnaround time for molecular detection of five main carbapenemases, *bla*_KPC_ (*Klebsiella pneumoniae* carbapenemase), *bla*_NDM_ (New Delhi metallo-β-lactamase), *bla*_IMP_ (Imipenemase), *bla*_VIM_ (Verona integron-encoded metallo-β-lactamase) and *bla*_OXA-48_ (oxacillin-hydrolysing), with higher sensitivity and reliability [1–4]. In 2019, the estimated number of deaths directly attributable to drug-resistant infections was 1.27 million, of which 70% was attributable to fluoroquinolones and β-lactam antibiotics like carbapenems [5]. In a cohort of Carbapeneamse-producing Enterobacterales (CPE) carriers, a recent study has showed a 24.1% incidence of CPE infection with a median time from detection of CPE carriage to infection of 15 days [6]. There is an urgent need for rapidly identifying patients at high risk of CPO infection for infection control and prevention.

A previous study on bronchial specimens investigated the correlation between bacterial count and cycle number (C_t_) to differentiate infections from colonisation [7]. A rapid assay to correlate and estimate carbapenemase-producing (CP) bacterial loads would aid future studies on bacterial load dynamics and allow us to determine how well bacterial load potentially predicts the risk of clinical infection. However, such studies are limited for stool samples. In this study, the correlation between Xpert Carba-R C_t_ values and colony counts of CP bacteria in stool samples was investigated.

## Material and methods

### Selection of clinical samples

De-identified stool samples were collected as part of an ongoing study investigating the household transmission of CPO in Singapore, reviewed and approved by the Domain Specific Review Board (DSRB) of National Healthcare Group Singapore (DSRB reference 2019/00794). Participants provided written informed consent. Patients from three multidisciplinary public hospitals were recruited and screened for CPOs. The recruitment period lasted from 1^st^ Feburary 2021 to 31^st^ January 2024.

Stool samples from CPO positive patients recruited in the above study were used to perform the validation experiments in this study. CPO positive stool samples were confirmed by a positive Xpert Carba-R run in conjuction with an 18 to 24-hour culture on the ChromID CARBA SMART (bioMérieux, Marcy-l’Étoile, France). Stool samples from CPO negative patients were used to perform the spiking experiments in this study. A CPO negative stool samples is defined as negative for both the Xpert Carba-R run and the ChromID CARBA SMART culture. Fresh stool samples were collected and stored at 4˚C immediately upon receipt, and transferred to -80˚C storage within 48 hours if not used immediately. For this study, only stool samples from participants who consented to storing their samples for future research were used.

### Sample preparation for Xpert Carba-R assay

To prepare stool suspensions, an Eswab (Copan Diagnostics, Murrieta, CA) was dipped into the stool sample and resuspended in Amies medium by vortexing for 10 seconds. We created a series of turbidity standards to standardise the amount of stool for each Xpert Carba-R assay run (S1 Appendix). Each Amies-stool suspension was prepared to match the most turbid standard. From the Amies-stool suspension, 300 μL was added into the Xpert sample reagent and vortexed [1]. From this mixture, 1.7 mL was transferred into an Xpert Carba-R cartridge and loaded on the GeneXpert platform according to the manufacturer’s instructions (Xpert Carba-R package insert; 301–2438, Rev. F).

### Generation of standard curve

Each CPO-negative stool sample was singly-spiked with a carbapenemase-producing organism carrying a single Xpert Carba-R target gene in its resistome. The isolates used were a strain of NDM-1-producing *Enterobacter cloacae*, IMP-1-producing *Pseudomonas aeruginosa*, KPC-2-producing *Escherichia coli*, VIM-2-producing *P*. *aeruginosa*, and OXA-48-producing *E*. *coli*. These isolates were previously obtained from purity plates of clinical or surveillance samples and stored in CryoCare vials (Key Scientific Products, Stamford, Texas) at -80˚C. For each gene, the experiment was repeated independently on three stool samples. The organisms were prepared in 0.9% sterile saline to a 0.5 McFarland standard (approximately 1 x 10^8^ cfu/mL) using a Densichek instrument (bioMérieux). Samples were then subjected to 10-fold serial dilutions to estimated concentrations of 10^1^, 10^2^, 10^3^, 10^4^, 10^5^, 10^6^, and 10^7^ cfu/mL in the Amies-stool suspension. Following which, the Xpert Carba-R assay was performed as described in previous section.

To determine bacterial counts, 100 μL of each sample was also inoculated on ChromID CARBA (bioMérieux) or ChromID OXA-48 (bioMérieux) plates in triplicates. Samples with estimated concentrations greater than 10^3^ cfu/mL were subjected to 10-fold serial dilutions to achieve 10^3^ cfu/mL suspensions, while the remaining samples were plated neat. Colonies were counted after incubation at 37˚C for 18 hours. As a negative control, an aliquot of the Amies-stool sample was loaded on the GeneXpert, as well as cultured on both ChromID CARBA and ChromID OXA-48 plates without prior spiking.

### Validation of standard curve

Stool samples from the ongoing study investigating the household transmission of CPO in Singapore that tested positive for carbapenemase gene by GeneXpert were used for validation. Based on the C_t_ value obtained, an estimated bacterial concentration was determined using the standard curves. The processed stool samples were diluted accordingly and plated on ChromID CARBA or ChromID OXA-48 plates in triplicates. The plate counts were recorded and compared with estimated values. We define delta values as the absolute difference between Carba-R estimated log(cfu/mL) and colony count log(cfu/mL). For each sample, delta values were recorded for evaluation. For samples that yielded multiple morphologies on ChromID CARBA or ChromID OXA-48 plates, GeneXpert was performed on each distinct morphology to check for CP status. Only those that were confirmed to be carbapenemase-producers were included in the counts.

### Statistical analysis

Regression lines showing the correlation between C_t_ values and final plate counts were plotted using Graphpad Prism. Statistical calculations to determine standard deviation and 95% confidence intervals of delta values were performed using Microsoft Excel.

## Results

### Evaluation of limit of detection for Xpert Carba-R assay

Runs with a C_t_ value but deemed analyte negative by the GeneXpert platform were recorded as negative. The highest C_t_ values measured for the five CPO isolates were, 36.9, 35.3, 37.7, 37.2 and 37.3 for the detection of *bla*_NDM_, *bla*_IMP-1_, *bla*_KPC_, *bla*_VIM_ and *bla*_OXA-48-type_, respectively, corresponding to plate counts of 1.88, 3.64, 1.87, 3.01 and 1.12 log(cfu/mL). *bla*_NDM_, *bla*_KPC_ and *bla*_OXA-48-type_ were consistently detected when spiked at an estimated concentration of 10^3^ to 10^7^ cfu/mL compared to *bla*_IMP-1_ and *bla*_VIM_, which were only consistently detected at estimated concentrations of 10^4^ to 10^7^ cfu/mL (S1 Table). The limit of detection (LOD), defined as the lowest concentration at which all repeat runs are positive [1], was higher for *bla*_IMP-1_ and *bla*_VIM_ (10^4^ cfu/mL) compared to *bla*_NDM_, *bla*_KPC_ and *bla*_OXA-48-type_ (10^3^ cfu/mL). All genes detected by the Xpert Carba-R assay correspond with the carbapenemase gene carried by the spiked organism; no false positives were detected in all samples.

**Fig 1:**
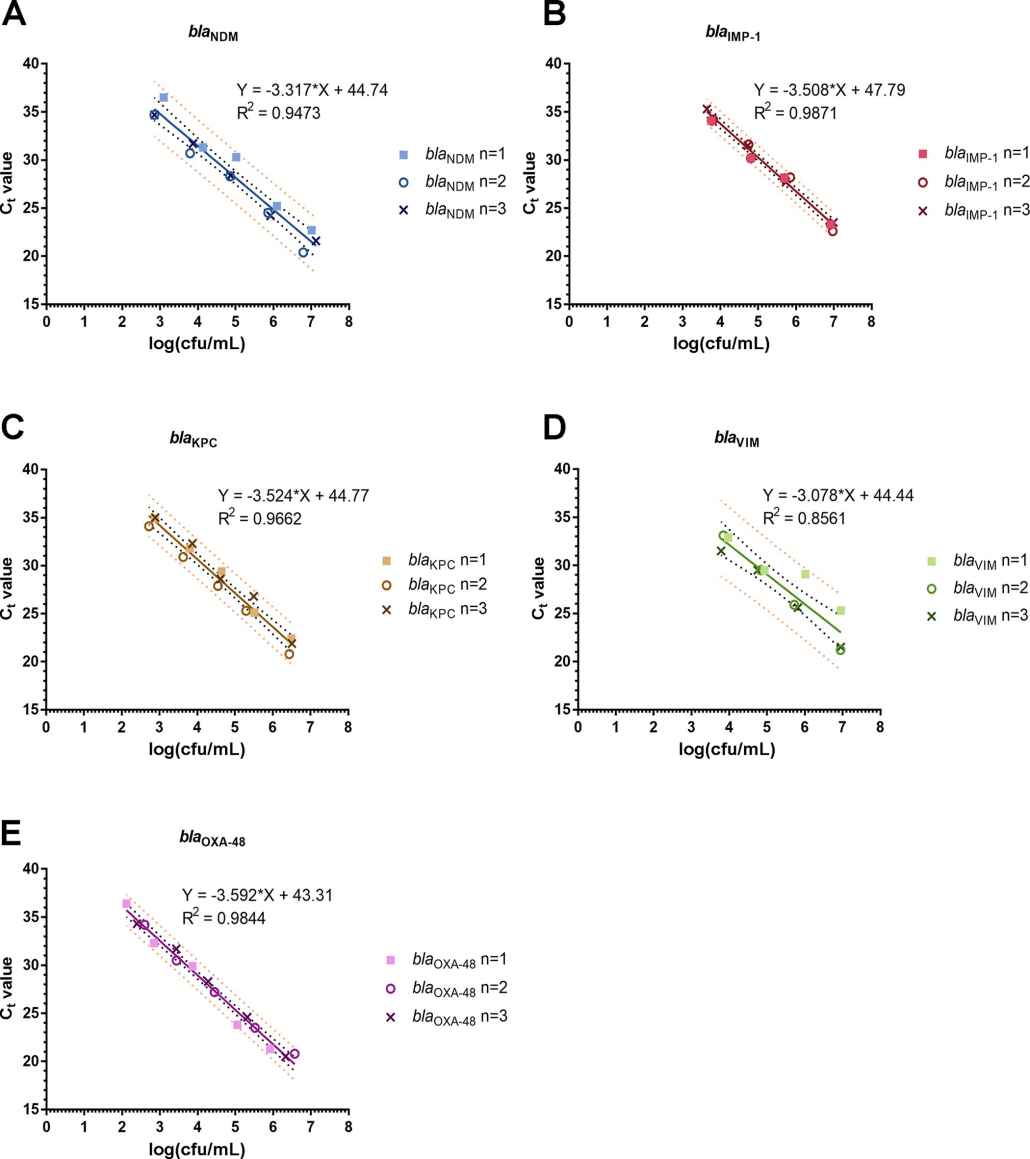
Standard curves of C_t_ values plotted against bacteria plate counts. (A-E) Linear regression lines of C_t_ values against log(cfu/mL) for stool samples spiked with NDM-1-producing *E*. *cloacae*, IMP-1-producing *P*. *aeruginosa*, KPC-2-producing *E*. *coli*, VIM-2-producing *P*. *aeruginosa*, and OXA-48-type-producing *E*. *coli* respectively. Black dotted lines denote 95% confidence interval while orange dotted lines denote 95% prediction interval.

A linear regression model was utilised to examine the correlation between C_t_ values and bacterial counts (Fig 1, Table 1). For each estimated concentration, only C_t_ values that were recorded on GeneXpert for all independent repeats were included in the analysis. The data points of all genes exhibited a good fit to a linear model with R^2^ values ranging between 0.8561 (*bla*_VIM_) and 0.9871 (*bla*_IMP-1_).

**Table 1:** Values used to plot standard curve.

CP Gene	n = 1		n = 2		n = 3	
	C_t_ value	log(CFU/mL)	C_t_ value	log(CFU/mL)	C_t_ value	log(CFU/mL)
*bla*_NDM_	36.5	3.093422	34.7	2.847161	34.7	2.853293
*bla*_NDM_	31.3	4.132473	30.7	3.792392	31.7	3.869232
*bla*_NDM_	30.3	5.01424	28.3	4.849215	28.4	4.861335
*bla*_NDM_	25.2	6.088727	24.5	5.857332	24.2	5.922552
*bla*_NDM_	22.7	7.011429	20.4	6.79005	21.6	7.126023
*bla*_IMP-1_	34.1	3.765917	34.2	3.808436	35.3	3.64015
*bla*_IMP-1_	30.2	4.801632	31.6	4.742987	31.5	4.721536
*bla*_IMP-1_	28.1	5.69314	28.2	5.845098	27.7	5.729705
*bla*_IMP-1_	23.3	6.913814	22.6	6.971585	23.5	6.99417
*bla*_KPC_	34.9	2.882714	34.1	2.718778	35	2.884607
*bla*_KPC_	31.8	3.80618	30.9	3.626682	32.3	3.871184
*bla*_KPC_	29.4	4.64673	27.9	4.548185	28.6	4.619789
*bla*_KPC_	25.1	5.50965	25.3	5.293731	26.8	5.496007
*bla*_KPC_	22.4	6.496007	20.8	6.436693	21.9	6.50515
*bla*_VIM_	32.9	3.970037	33.1	3.843025	31.5	3.78533
*bla*_VIM_	29.5	4.934498	29.5	4.838849	29.5	4.750765
*bla*_VIM_	29.1	6.018423	25.9	5.718778	25.6	5.817345
*bla*_VIM_	25.3	6.952631	21.2	6.946125	21.5	6.955848
*bla*_OXA-48_	36.4	2.113943	34.2	2.598426	34.3	2.39794
*bla*_OXA-48_	32.3	2.845098	30.5	3.441957	31.7	3.436693
*bla*_OXA-48_	29.9	3.865301	27.2	4.447158	28.3	4.278754
*bla*_OXA-48_	23.8	5.054358	23.5	5.518514	24.6	5.31527
*bla*_OXA-48_	21.3	5.920819	20.8	6.572097	20.5	6.322219

### Samples for validation of standard curve

The standard curves were validated using selected stool samples obtained from an ongoing study investigating the household transmission of CPO in Singapore. The samples used for validation were collected between 7^th^ March and 13^th^ July 2022 and only carbapenamase-positive samples were used. Among 133 total patient stool samples, seven *bla*_NDM_, one *bla*_IMP-1_ and eleven *bla*_OXA-48-type_ samples were used for validation.

### Evaluation of standard curve

Bacterial counts of stool samples were quantified by culture and compared to estimated values determined from the standard curve (Fig 1). C_t_ values, Carba-R estimated bacterial counts and colony counts determined from traditional culture are summarised in Table 2. Delta values, defined as the absolute difference between Carba-R estimated log(cfu/mL) and colony count log(cfu/mL), were also calculated. A delta value of 0.71 log(cfu/mL) was observed for the sole *bla*_IMP-1_ sample, while a maximum delta value of 1.36 log(cfu/mL) and 1.56 log(cfu/mL) was observed for *bla*_NDM_ and *bla*_OXA-48-type_, respectively (Table 2). Statistical analysis was only performed for *bla*_NDM_ and *bla*_OXA-48-type_ samples as only one *bla*_IMP-1_ sample and no *bla*_KPC_ and *bla*_VIM_ samples were received during the study period. The average delta value for *bla*_NDM_ and *bla*_OXA-48-type_ was 0.56 log(cfu/mL) (95% CI 0.24–0.88) and 0.80 log(cfu/mL) (95% CI 0.53–1.07), respectively (S2 Table), suggesting that the standard curve of *bla*_NDM_ was able to predict bacterial loads more accurately than that of *bla*_OXA-48-type_. However, due to the small sample size, the accuracy of the standard curves cannot be conclusively defined.

**Table 2:** Summary of Ct values, Carba-R estimated and colony counted bacterial loads of clinical samples.

Sample no.	CP Gene	C_t_ value	Carba-R estimated (cfu/mL)	Colony Count (cfu/mL)	Delta value^a^
1	*bla*_IMP-1_	27.60	5.76	5.04	0.71
2	*bla*_NDM_	27.20	5.29	4.60	0.69
3	*bla*_NDM_	20.80	7.22	6.56	0.66
4	*bla*_NDM_	30.00	4.44	4.33	0.11
5	*bla*_NDM_	22.30	6.77	6.28	0.49
6	*bla*_NDM_	23.30	6.46	6.43	0.032
7	*bla*_NDM_	33.60	3.36	4.72	1.36
8	*bla*_NDM_	29.00	4.75	5.33	0.58
9	*bla*_OXA-48_	24.70	5.18	5.85	0.67
10	*bla*_OXA-48_	29.10	3.96	2.51	1.45
11	*bla*_OXA-48-type_ ^b^	22.40	5.82	4.83	0.99
12	*bla*_OXA-48_	31.40	3.32	3.15	0.17
13	*bla*_OXA-48_	22.40	5.82	4.99	0.84
14	*bla*_OXA-48_	21.10	6.18	5.97	0.21
15	*bla*_OXA-48_	22.90	5.68	4.12	1.56
16	*bla*_OXA-48_	25.60	4.93	5.88	0.95
17	*bla*_OXA-48-like_	30.70	3.51	4.31	0.80
18	*bla*_OXA-48_	28.10	4.23	3.40	0.83
19	*bla*_OXA-48_	25.40	4.99	4.66	0.33

^a^ Absolute difference between colony-counted bacterial load and Carba-R estimated values determined from standard curves

^b^ *bla*_OXA-48-type_ includes *bla*_OXA-48_ and *bla*_OXA-48-like_ CP genes

## Discussion

The GeneXpert platform has been widely reported for its sensitivity and specificity [1–4], and would be a good test to correlate C_t_ values and bacterial loads. Here, using spiked stool samples, standard curves were generated to estimate bacterial loads based on C_t_ values measured by GeneXpert. We were able to estimate bacterial counts for *bla*_NDM_ and *bla*_OXA-48-type_ samples with errors of 0.56 log(cfu/mL) (95% CI 0.24–0.88) and 0.80 log(cfu/mL) (95% CI 0.53–1.07), respectively. We could not validate the other three genes as *bla*_IMP-1_-positive stool samples were rare, while there were no *bla*_KPC_-positive and *bla*_VIM_-positive samples during the study period. We noted a lower LOD across all genes compared to what Yee and colleagues have shown, despite using a similar preparation method. This could be attributed to differences in the methods as to how the stool samples were spiked [1].

To our knowledge, besides Burillo’s group [7], there are no studies evaluating the performance of the Carba-R assay for predicting bacterial or gene loads. Burillo *et al*. showed that based on C_t_ values, they could determine if the bacterial load in a bronchial sample was ≥10^5^ cfu/mL (C_t_ ≤ 24.7), ≥10^4^ cfu/mL (24.7 < C_t_ ≤ 26.9), or <10^4^ cfu/mL (C_t_ > 26.9) [7]. Similarly, our study shows the correlation of C_t_ values and bacterial loads, but in stool samples. Additionally, in a previous study by Ko *et*. *al*. to evaluate the diagnostic performance of the Carba-R assay using rectal swabs, a regression line was generated to compare GeneXpert C_t_ values and cultures of carbapenemase resistant organisms [4]. However, as the purpose of Ko’s group’s study was not to estimate bacterial loads, only one line was plotted for all five genes [4]. Here, we show that there are slight differences in Carba-R assay’s analytical sensitivity for detecting each gene. Notably, in accordance with the higher LOD obtained for *bla*_IMP_ and *bla*_VIM_, each C_t_ value also corresponded with a higher bacterial count compared to *bla*_NDM_, *bla*_KPC_ and *bla*_OXA_. While there are few studies that verify the LOD of each gene target, higher LODs for *bla*_IMP-1_ and *bla*_VIM_ have been reported [4, 8]. This may reflect lower copy numbers of these two genes as compared to *bla*_KPC_, *bla*_NDM_ and *bla*_OXA-48-type_. This should be taken into account when the standard curve is used for bacterial load estimation (Fig 1 and S1 Table). If implemented, the inclusion of a variety of isolates should be considered to improve the robustness of the standard curve. We also noted that the standard curve of *bla*_NDM_ was able to predict bacterial loads more accurately than that of *bla*_OXA-48-type_. Of eleven *bla*_OXA-48-type_*-*containing stool samples, three had Carba-R estimated loads that were greater than 1 log(cfu/mL) above the bacterial counts from culture. OXA-48-type enzymes are known for being weakly hydrolysing, especially so if the OXA-48-type producing isolate do not produce ESBL [9, 10]. This can hinder growth on selective media and may have led to an underestimation of the true bacterial load in the three aforementioned samples. Again, this highlights the limitations of culture-only methods for detection and quantification of CPO.

Patients with intestinal colonisation of pathogens, notably *Klebsiella pneumoniae*, are at higher risk of developing clinical infections [11, 12]. This applies to CPO as well; patients who developed CPO infections were associated with higher relative load of KPC or OXA-48 producing bacteria in the gut [12–14]. The use of GeneXpert C_t_ values could facilitate quicker identification of patients at higher risk of clinical infections for early treatment interventions.

Heterogeneity in infectious disease dynamics, where a small proportion of individuals is responsible for a large proportion of transmission events [15], has been described for several pathogens including *E*. *coli* [16] and *K*. *pneumoniae* [17]. Infectiousness is most simply and frequently measured by bacterial load in samples such as feces [15, 18]. Multiple studies have shown that patients with higher bacterial loads were more likely to be capable of contaminating the environment and the personal protective equipment of nursing staff [19–22], which are known to be a reservoir and source of transmission of CPOs [23–27]. A recent study in a mouse model suggested that individuals who shed higher loads of bacteria are the main contributors to host-to-host transmission events [17]. Early identification and cohorting of colonized patients and nursing staff is one of the only, if not the only, ways of preventing nosocomial CPO outbreaks [28, 29], and infection control protocols that do not reach the individuals with high bacterial loads may be inadequate [15, 30]. However, cohorting is costly and often difficult to implement [26]. When resources are limited, the ability to estimate bacterial loads based on GeneXpert C_t_ values could allow for prioritisation of infection prevention strategies, such as decolonisation interventions and cohorting to minimize environmental contamination. Aside from clinical benefits, this would also serve as a more efficient method to estimate bacterial or gene loads for load dynamics studies in future research.

This study has several limitations. When generating a standard curve for each carbapenemase gene, the negative stool samples were spiked with a single organism. Further large-scale studies with more diverse clinical samples would ensure greater representation and account for potential variation between bacterial strains and species. In Singapore, CPE isolates collected from 2010 to 2015 across multiple hospitals revealed that the most prevalent carbapenemase genes were *bla*_KPC_, followed by *bla*_NDM_, *bla*_OXA48-type_, and *bla*_IMP_ [31]. For the duration of the current study, subject recruitment from other hospitals was still a work in progress; however, once implemented, would help to address the current lack of *bla*_KPC_ samples and allow a better representation of the carbapenemase gene distribution in Singapore. The standard curve was also generated based on the assumption that the CPO carries a single copy gene. While isolates carrying multiple carbapenemase gene copies have been reported [32, 33], the overall frequency of these cases is unknown. This should not be a major limitation as it was previously shown that gene loads were linearly correlated to their host strains’ abundance [12]. The applicability of the curve for predicting infection risk will also not be undermined as higher carbepenemase gene loads are also associated with higher risk of infection [13, 34]. Moreover, quantification of plasmid copy numbers requires DNA from pure cultures which require multiple days to obtain [35]. In times of large outbreaks, such methods may be too labour- and time-intensive, and assays with a short turnaround time for identification of high risk patients at the cost of a reasonable amount of accuracy may be favourable. However, in a non-outbreak setting with less time constraints, more comprehensive methods to determine bacterial loads may be employed instead.

Where appropriate validation is performed for the population of interest, this method enables the estimation of bacterial loads in the same turnaround time as the GeneXpert Carba-R assay without the need for traditional methods. Traditionally, to quantify the relative load of CPO or carbapenemase genes, the ratio of cultured CPO to total viable aerobic bacteria or the 2^-ΔΔCt^ method, respectively, had to be used [12, 13, 36]. Using our standard curve, the same could be achieved within an hour, allowing research decisions to be made rapidly. Additionally, this assay could aid studies like bacterial load dynamics and correlation of bacterial loads to persistent CPO carriage, which could potentially be applied in clinical settings in the future.

## Supporting information

S1 Appendix Turbidity standards. All stool-amies suspensions were adjusted to 1x dilution.
(TIF)

S1 Table Summary of positive runs for each carbapenemase gene at each estimated concentration. (DOCX)

S2 Table Validation of *bla*_NDM_ and *bla*_OXA-48_ standard curves. (DOCX)
