# Supplementary figures and images for: Evaluation of the Xpert Carba-R assay for quantifying carbapenemase-producing bacterial load in stool samples
Source: PLoS One. 2024 Aug 28;19(8):e0309089. doi: 10.1371/journal.pone.0309089 (PMC11356397; doi:10.1371/journal.pone.0309089)

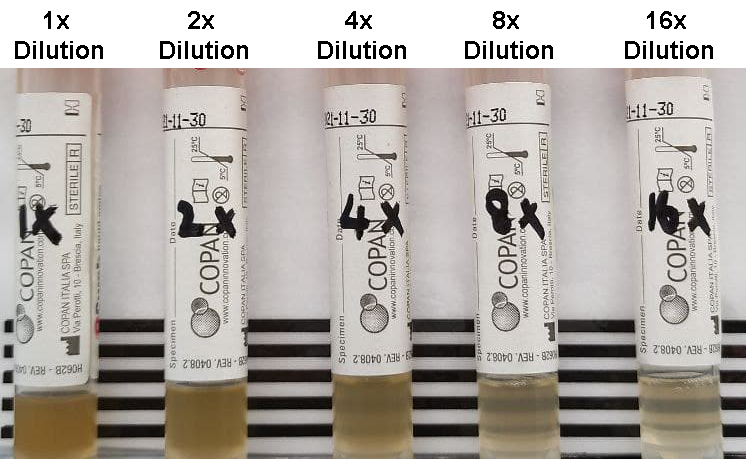

Supplement: S1 Appendix — All stool-amies suspensions were adjusted to 1x dilution. (TIF) [file pone.0309089.s001.tif]
