# Supplementary material for: Evaluation of the Xpert Carba-R assay for quantifying carbapenemase-producing bacterial load in stool samples
Source: PLoS One. 2024 Aug 28;19(8):e0309089. doi: 10.1371/journal.pone.0309089 (PMC11356397; doi:10.1371/journal.pone.0309089)
Supplement: S1 Table — (DOCX) [file pone.0309089.s002.docx]

**S1 Table: Summary of positive runs for each carbapenemase gene at each estimated concentration**

| **CP gene** | **No. of runs** | **Estimated cfu/mL** | | | | | | |
| --- | --- | --- | --- | --- | --- | --- | --- | --- |
|  |  | **10**^7^ | **10**^6^ | **10**^5^ | **10**^4^ | **10**^3^ | **10**^2^ | **10**^1^ |
| *bla*_NDM_ | 3 | 3 | 3 | 3 | 3 | 3 | 1 | 0 |
| *bla*_IMP-1_ | 3 | 3 | 3 | 3 | 3 | 0 | 0 | 0 |
| *bla*_KPC_ | 3 | 3 | 3 | 3 | 3 | 3 | 1 | 0 |
| *bla*_VIM_ | 3 | 3 | 3 | 3 | 3 | 1 | 0 | 0 |
| *bla*_OXA-48_ | 3 | 3 | 3 | 3 | 3 | 3 | 2 | 0 |
