# Supplementary material for: Evaluation of the Xpert Carba-R assay for quantifying carbapenemase-producing bacterial load in stool samples
Source: PLoS One. 2024 Aug 28;19(8):e0309089. doi: 10.1371/journal.pone.0309089 (PMC11356397; doi:10.1371/journal.pone.0309089)
Supplement: S2 Table — (DOCX) [file pone.0309089.s003.docx]

**S2 Table: Validation of bla_NDM_ and bla_OXA-48_ standard curves**

| **CP Gene** | **No. of samples** | **Average delta value** | **Standard deviation of delta values** | **95% CI of delta values** |
| --- | --- | --- | --- | --- |
| *bla*_NDM_ | 7 | 0.56 | 0.44 | 0.32 |
| *bla*_OXA-48_ | 11 | 0.80 | 0.45 | 0.27 |
